# Light affects in vitro organogenesis of *Linum usitatissimum* L. and its cyanogenic potential

**DOI:** 10.1007/s11738-012-1118-4

**Published:** 2012-10-12

**Authors:** Irena Siegień, Aneta Adamczuk, Katarzyna Wróblewska

**Affiliations:** Institute of Biology, The University of Bialystok, Świerkowa 20b, 15-950 Bialystok, Poland

**Keywords:** Cyanogenesis, Exogenous hormones, Flax, Linamarin, Regeneration

## Abstract

The relationships between organogenesis of oil flax (*Linum usitatissimum* L., cv. ‘Szafir’) in vitro, cyanogenic potential (HCN-p) of these tissues and light were investigated. Shoot multiplication obtained on Murashige and Skoog medium containing 0.05 mg L^−1^ 2,4-dichloro-phenoxyacetic acid and 1 mg L^−1^ 6-benzyladenine (BA), was about twice higher in light-grown cultures than those in darkness. Light-grown explants showed also higher rate of roots regeneration (in medium containing 1 mg L^−1^ α-naphtaleneacetic acid and 0.05 mg L^-1^ BA) than dark-grown ones. The cyanogenic potential (expressed both as linamarin and lotaustralin content and linamarase activity) of flax cultured in vitro was tissue-specific and generally was higher under light conditions than in darkness. The highest concentration of linamarin and lotaustralin was detected in light-regenerated shoots, and its amount was twice as high as in roots, and about threefold higher than in callus tissue. The activities of linamarase and β-cyanoalanine synthase in light-regenerated organs were also higher than those in darkness. Thus, higher frequency of regeneration of light-grown cultures than dark-grown ones seems to be correlated with higher HCN-p of these tissues. We suggest that free HCN, released from cyanoglucosides potentially at higher level under light conditions, may be involved in some organogenetic processes which improve regeneration efficiency.

## Introduction

The species *Linum usitatissimum* L. (flax) has been the focus of a great deal of both applied and basic research effort in plant cell and biotechnology studies in recent years. It is the oldest cultivated plant and is the only member of Linaceae that is of economic importance—source of fibre and oil. The oily form (linseed) of *L. usitatissimum* is a significant crop plant, particularly in cool temperate environments, with an annual world production of around three million metric tonnes (Millam et al. 2005). Linseed crops are grown mainly for the seed oil, used as a source of industrial oil. In recent years the increasing interest in health products has highlighted this species as being a significant source of plant omega-3 fatty acids (Abbadi et al. 2004). Furthermore, post-harvest the linseed crop residue can be crushed and used as a valuable feed for livestock.

*Linum usitatissimum* is a cyanogenic plant, known to contain in vegetative tissues two cyanogenic glucosides—linamarin and lotaustralin (Cutler et al. 1985). There are monoglucosides in which the aglycone moieties are the cyanohydrins of acetone and 2-butanone, respectively, and the glycon moiety is d-glucose attached as an O-β-glucosyl linkage. In addition flax seeds contain two cyanogenic diglucosides, linustatin and neolinustatin, the 6′-*o*-glucosides of linamarin and lotaustralin, respectively. The cyanogenic potential (capacity to produce hydrogen cyanide—HCN-p), (Lieberei et al. 1986) changes during germination, seedling development and subsequent growth of flax plants (Niedźwiedź-Siegień 1998). The total amount of cyanogens increased markedly during early seedlings growth and, before the onset of flowering. Monoglucosides were found mainly in leaves of flax plants throughout the whole vegetation period, but the highest concentration was characteristic of flowers. In contrast, these glucosides occured in relatively small amounts in roots and in stems (Niedźwiedź-Siegień 1998).

The degradation of cyanogenic glycosides starts with removal of sugar moiety by the action of specific β-glycosidases (EC. 3.2.1.-). The resulting cyanohydrins are relatively unstable and decompose either spontaneously or enzymatically in reaction catalysed by α-hydroxynitrile lyase (EC. 4.1.2.-) to an aldehyde or a ketone, and free HCN (Vetter 2000). HCN is toxic to aerobic organisms as an inhibitor of respiration and heavy metals containing enzymes. β-Cyanoalanine synthase (β-CAS), (EC.4.4.1.9), plays pivotal role in detoxification of cyanide. It catalyses the reaction of cysteine and cyanide to form hydrogen sulphide and β-cyanoalanine. The latter is subsequently converted to asparagine in reaction catalysed by β-cyanoalanine hydrolase (EC. 4.2.1.65), (Wurtele et al. 1984).

The roles of cyanogenic glycosides and HCN vary, and are understood only to some extent (Møller 2010). They are important factors of plant defence systems against herbivores, insects and pathogens (Gleadow and Woodrow 2002). Cyanoglycosides may also play an important role in the primary metabolism and could function as nitrogen containing precursors for amino acid and protein synthesis during seedling development (Lieberei et al. 1986; Sanchez-Perez et al. 2009). Moreover, free cyanide, including the one released from the cyanogenic glycosides, may act as signalling molecule (Siegień and Bogatek 2006; Oracz et al. 2009; Gniazdowska et al. 2010).

Many recent reports showed protocols for regeneration of flax and propagation in vitro (e.g. Gomes da Cunha and Ferreira 1996; Dedičová et al. 2000; Rutkowska-Krause et al. 2003). Some data indicate that induction of organogenesis of *Linum* in vitro is a stress-related response (Verdus et al. 1997; Mundhara and Rashid 2001; Obert et al. 2005). Recently, Kaira and Babbar (2010) reported the promotory effect of nitric oxide (NO) donors on shoot organogenesis from hypocotyl explants of flax. Moreover, some organogenetic processes may be differently affected by light (Liu et al. 1983; Mundhara and Rashid 2002; Hassanein and Dorion 2005). The formation of many plant primary and secondary metabolites is also light-controlled (Gaisser and Heide 1996; Liu et al. 2002), and accumulation of cyanogenic glucosides was observed mainly in flax seedlings in light (Niedźwiedź-Siegień and Giersimiuk 2001). It was also detected (Niedźwiedź-Siegień 1998), that the cyanogenic potential of intact flax plants, as other cyanogenic plants (Kojima et al. 1979) is tissue specific, and thus related to tissue differentiation.

Main objective of this study was to investigate the relationship between organogenesis of flax in vitro and the cyanogenic potential of these tissues, which seems to be under control of light. The working hypothesis was that HCN emission resulting from light-controlled hydrolysis of cyanogenic glucosides may trigger some events leading to tissue differentiation.

## Materials and methods

### Plant material, in vitro germination

Seeds of oil flax (*L. usitatissimum* L., cv. ‘Szafir’) harvested in 2007 were obtained from Plant Breeding and Acclimatization Institute Strzelce (Poland), and stored dry at 4 °C in darkness for 6–15 months before experiments. Seeds were immersed in 70 % ethanol for 1 min rinsed twice in sterile distilled water and surface sterilized in 6 % sodium hypochlorite solution (6 % available chlorine) for 20 min with a drop of Tween-20 per 100 mL. Then seeds were rinsed four times in sterile distilled water and sown in 9-cm-diameter Petri dishes filled with 0.75 % agar (Sigma). Seeds were cultured for 7 days in a growth chamber under 16-h light photoperiod (described in text as “light”), day/night temperature 25/20 °C, light intensity 50 μmol m^−2^ s^−1^ (provided by cool white fluorescent tubes). For dark germination, Petri dishes with sterile seeds were placed into growing chamber in black boxes.

### Media and culture conditions

The basal medium contained Murashige and Skoog (MS) mineral salts and vitamins (Murashige and Skoog 1962), 3 % (w/v) sucrose, 0.75 % (w/v) agar (Sigma). Media 1, 2 and 3 (abbreviated as M1, M2 and M3) were prepared by supplementation of basal MS with plant growth regulators (PGRs) as follows:

M1: MS + 2,4-dichloro-phenoxyacetic acid (2,4-D, 1 mg L^−1^) and 6-benzyladenine (BA, 0.75 mg L^−1^);

M2: MS + 2,4-D (0.05 mg L^−1^) and BA (1 mg L^−1^);

M3: MS + α-naphtaleneacetic acid (NAA, 1 mg L^−1^) and BA (0.05 mg L^−1^).

M1, M2 and M3 media were prepared as described by Yildiz and Özgen (2004) and Dedičová et al. (2000) with some modifications. Before autoclaving (121 °C, 101.3 kPa, 25 min) pH of the media was adjusted to 5.8 with 1 M KOH. The media were aliqued to Petri dishes (Ø 90 mm) 30 mL each. Culture was prolonged in light or dark under the same conditions as described for seed germination.

### Effect of light and PGRs on morphogenic responses and biomass accumulation

Explants excised from medial parts of hypocotyls of light-grown seedlings (4–5 mm, one piece from hypocotyl) were cultured in Petri dishes (10 explants per dish, 4 dishes per treatment) on M1, M2 and M3 medium in light or in darkness for 4 weeks. Then, the morphogenic response, expressed as callus induction, shoots and roots regeneration was detected. To measure tissue fresh mass, at least 12 explants, typical for each culture, were weighed; then explants were dried to constant weight at 105 °C, and their dry mass was determined.

### Effect of light conditions of donor plants on shoot or root organogenesis

To study the effect of light conditions of donor plants on explants morphogenic response, parts of hypocotyls derived from light- or dark-grown seedlings were used. Both groups of explants, prepared as described above, were cultured on M2 (shoot organogenesis) or M3 medium (root organogenesis), in light or in darkness. The morphogenic response, expressed as percent of explants with shoots or roots, and the number of shoots or roots on appropriate explant, after 2 and 4 weeks of culture growth were detected.

### Morphogenic response expression

The percentage of explants with shoots (on M2) or roots (on M3) and average number of shoots or roots per responsive explants was calculated according to the formula (Zhao et al. 2007):

Percentage of explants with a shoots or roots (%)

$$ = \frac{{ {\text{Number\; of\; explants\; with\; shoots\; or\; roots\;}}}}{\text{Total number of callused explants}} \times 100 $$

Average number of shoots or roots per responsive explant

$$ = \frac{\text{Sum\; of\; the\; number\; of\; shoots\; or\; roots\; in\; each\; explant}}{{ {\text{Number of explants with shoots or roots}}}} \times 100 $$

### Cyanoglucoside content, linamarase and β-CAS activities

Cyanogenic glucoside content and activities of linamarase and β-CAS were determined in shoots and roots of 7-day-old seedlings of flax, and in 4-week-old tissues (callus, regenerated shoots and roots) originated from light-grown seedlings.

#### Cyanogenic glucosides determination

Cyanogenic glucosides (linamarin-2-hydroxyisobutyronitrile β-d-glucopyranoside, and lotaustralin-2-hydroxy-2-methylbutyronitrile β-d-glucopyranoside) were extracted from fresh plant material, separated by thin layer chromatography (TLC) and determined as described previously (Niedźwiedź-Siegień 1998). Cyanogenic glucosides were detected on chromatograms by a sandwich technique with Feigl-Anger testpaper (Kakes 1991). The appearance of two blue spots on the Feigl-Anger paper was observed every time. One of the spots was identified as linamarin (*R*_f_ = 0.59), based on *R*_f_ of linamarin standard (Sigma). Another one was identified as lotaustralin, (*R*_f_ = 0.64), based on the results described by Hahlbrock and Conn (1971) and Cutler et al. (1985). Moreover, these localizations were consistent with our previous data (Niedźwiedź-Siegień 1998).

#### Extraction of crude β-glucosidase

Crude extract of β-glucosidase from flax seeds for cyanogen visualization on chromatograms and for hydrolysis of cyanogenic glucosides eluted from chromatograms was prepared and assayed as described previously (Niedźwiedź-Siegień 1998; Siegień 2009).

#### Linamarase activity determination

Linamarase assay was done using 5 mM linamarin diluted in 50 mM sodium citrate buffer, pH 6.0 (Joseph et al. 1999). The reaction was carried out for 15 min at 37 °C, and the glucose released was determined using glucose oxidase reagent (Yeoh 1989). Enzyme activity was expressed as nmol of glucose released within 1 s per 1 mg of protein (nkat).

#### β-Cyanoalanine synthase (β-CAS) activity determination

β-CAS assay was based on the reduction of methylene blue by H_2_S, produced during β-cyanoalanine synthesis in reaction of cysteine and HCN (Elias et al. 1997). The results are expressed in nmol of H_2_S released within 1 s by 1 mg of protein (nkat).

### Soluble protein determination

Protein was quantified by Bradford’s method (Bradford 1976) using bovine serum albumin (Sigma Aldrich) as a protein standard.

### Statistical analyses

All regeneration experiments were done using 10 explants per plate with at least four replications per treatment and were repeated at least twice. For enzymes' activities, at least four biological replicates were prepared, and experiments were done at least twice.

The results are presented as mean values ± SD. Means between the treatments were compared at the 0.05 probability level (using *t* Student test).

## Results

### Effects of light and PGRs on the morphogenic response of explants, and biomass accumulation

The morphogenic responses of flax explants, derived from light-grown seedlings, varied depending on PGRs concentration in the media (M1, M2, and M3) (Table 1). When BA concentration in the medium was slightly lower than 2,4-D (M1), only callus was formed. Explants produced mainly shoots when BA content strongly exceed 2,4-D, (M2). However, roots were formed in M3 medium with substantially higher NAA concentration compared to BA one. Formation of shoots or roots, respectively, on M2 or M3 medium was accompanied also by production of callus tissue at the cut slices of explants. The rate of shoot regeneration in the presence of light was about 30 % higher than the one obtained in darkness, whereas the rhizogenic processes under both conditions were similar. Fresh and dry masses of light-grown cultures were about twice higher than those from darkness. These parameters were the highest for cultures on M2 but the lowest for callus formed on M1 (Table 1).

**Table 1:** Effects of light and PGRs on the morphogenic responses, fresh and dry mass of light-derived explants of *L. usitatissimum*, cultured in vitro for 4 weeks

Medium	Growth regulator (mg L^−1^)		Regeneration	Morphogenic response (% of explants)		Fresh mass (mg/explant)		Dry mass (mg/explant)	
				Dark	Light	Dark	Light	Dark	Light
M1	BA	0.75	Callus	100^aA**^	100^aA^	114.7 ± 22.6^bB^	185.0 ± 27.5^cA^	8.8 ± 1.2^cbB^	15.9 ± 3.4^cA^
	2,4-D	1.0							
M2	BA	1.0	Shoots	65.2 ± 8.7^bB*^	100^aA^	186.0 ± 32.5^aB^	410.0 ± 47.0^bA^	13.0 ± 2.1^abB^	33.7 ± 3.2^aA^
	2,4-D	0.05							
M3	BA	0.05	Roots	67.7 ± 6.5^bB^	81.0 ± 8.5^bA^	133.0 ± 25.9^abB^	241.0 ± 26.7^bA^	10.6 ± 2.6^abB^	22.3 ± 3.1^bA^
	NAA	1.0							

* Mean values ± SD

** Means with the same lower case letter within a column are not significantly different while means with the same capital letter within a row are not significantly different (*P* = 0.05)

### Effect of light conditions of donor plants on shoot or root organogenesis

Light- and dark-derived explants cultured under light conditions for 2 weeks differed in the rate of organogenesis. Regeneration of shoots and roots from light-derived explants was about 30 % higher than from that from dark-derived ones. However, the average number of shoots or roots per one explant coming from light- or dark-grown seedlings, was similar (Table 2). The significant differences in shoots and roots regeneration were observed in explants excised from light- or dark-grown seedlings of flax cultivated for 2 weeks in darkness. Under these conditions, shoot regeneration on light-derived explants was noticeable (about 40 %), whereas there were no shoots or roots on dark-derived ones.

**Table 2:** Effect of light on shoot and root organogenesis from *L. usitatissimum* hypocotyl explants derived from light- and dark-grown seedlings

Medium	Organogenesis	Light-derived explants				Dark-derived explants			
		Light-cultivated		Dark-cultivated		Light-cultivated		Dark-cultivated	
		2 weeks	4 weeks	2 weeks	4 weeks	2 weeks	4 weeks	2 weeks	4 weeks
M2	Shoot								
	Shoot formation (%)	84.0 ± 3.2^c*^	100^a**^	40.0 ± 4.5^f^	66.9 ± 4.6^d^	53.3 ± 7.4^e^	93.0 ± 4.0^b^	0^h^	19.0 ± 9.0^g^
	Shoots/explant	4.0 ± 0.8^b^	10.0 ± 1.4^a^	2.4 ± 1.0^b^	4.9 ± 2.1^b^	3.1 ± 1.7^b^	8.8 ± 2.3^a^	0^c^	2.5 ± 1.2^b^
M3	Root								
	Root formation (%)	35.0 ± 10.2^b^	80.0 ± 7.8^a^	3.2 ± 1.7^d^	71.0 ± 8.3^a^	10.3 ± 3.2^c^	76.7 ± 7.2^a^	0^e^	26.4 ± 10.2^b^
	Roots/explant	3.2 ± 1.0^b^	13.5 ± 4.9^a^	3 ± 1.4^b^	4.7 ± 1.7^b^	4.3 ± 1.3^b^	11.7 ± 3.8^a^	0^c^	3.0 ± 1.6^b^

* Mean ± SD

** Means with the same letter within a row are not significantly different (*P* = 0.05)

Explants excised from light- or dark- grown seedlings, cultured under light conditions showed the similar rate of regeneration, expressed as percentage of explants with shoots or roots in 4-week-old cultures. The average number of shoots and roots regenerated per one explant under light conditions was also nearly the same, regardless of the type of explants (Table 2). However, the significant reduction of organogenesis was observed when light- and, especially dark-derived explants were cultivated in darkness. Efficiency of shoot or root regeneration was three times lower in the case of dark-derived explants cultivated in darkness compared with light-derived explants cultivated under the same conditions. Of course, the percentage of light-derived explants with shoots cultured in darkness was about 30 % lower than the one cultured in light (Table 2), as it was mentioned earlier (Table 1). The number of shoots or roots per explant was twice higher for light grown cultures than for dark ones, regardless of light conditions of flax seedling used as explants donor.

### Cyanogenic glucosides content, linamarase and β-cyanoalanine synthase activities under dark and light conditions

Shoots of flax, both seedling-derived and regenerated in vitro from hypocotyl explants, showed significant differences in accumulation of linamarin and lotaustralin, depending on light conditions. In seedling-derived shoots grown in light, linamarin and lotaustralin levels were higher than in dark-grown ones (Table 3). Similarly, the concentration of these cyanoglucosides in light-regenerated shoots was about twice higher than that in darkness. Roots, however, accumulated these compounds at low, but similar levels, regardless of light conditions. Low amount of cyanogenic glucosides was also detected in callus tissue, but only under light conditions (Table 3).

**Table 3:** Effects of light on cyanogenic glucosides content, linamarase and β-CAS activities in organs of 7-day-old *L. usitatissimum* seedlings at time explanation, and in tissues (callus, shoots, roots) regenerated in vitro

Tissue	Cyanogenic glucosides (mg g^−1^ fresh mass)		Linamarase (nkat mg^−1^ prot.)		β-CAS (nkat mg^−1^ prot.)	
	Dark	Light	Dark	Light	Dark	Light
Seedling						
Shoot	3.0 ± 0.4^aB*^	4.8 ± 0.8^aA**^	1.12 ± 0.1^bB^	1.94 ± 0.2^aA^	0.19 ± 0.02^cB^	0.27 ± 0.02^dA^
Root	1.1 ± 0.1^bcA^	1.3 ± 0.2^cA^	0.83 ± 0.12^cA^	0.65 ± 0.06^bA^	0.12 ± 0.03^dB^	0.21 ± 0.03^eA^
In vitro***						
Callus	0^dB^	0.8 ± 0.36^cA^	0.23 ± 0.06^eA^	0.21 ± 0.07^cA^	0.65 ± 0.14^aA^	0.88 ± 0.18^aA^
Shoot	1.4 ± 0.3^bB^	3.2 ± 0.3^bA^	0.46 ± 0.1^dB^	0.80 ± 0.09^bA^	0.17 ± 0.03^cdB^	0.35 ± 0.02^cA^
Root	0.8 ± 0.2^cA^	1.2 ± 0.3^cA^	1.43 ± 0.08^aB^	1.80 ± 0.02^aA^	0.30 ± 0.03^bB^	0.49 ± 0.05^bA^

* Means ± SD

** Means with the same lower case letter within a column are not significantly different while means with the same capital letter within a row are not significantly different (*P* = 0.05)

*** Callus induction, shoot and root organogenesis were obtained in M1, M2 and M3 mediums, respectively

The activity of HCN-liberating β-glucosidase in flax in vitro, determined against synthetic linamarine, was differently influenced by light depending on tissue. As in the case of cyanoglucoside content, specific activity of this enzyme in shoots (designated as linamarase according to Collinge and Hughes 1982) was almost twice higher under light conditions than that in darkness. Enzyme activity was also enhanced in light-regenerated roots, as compared to those from darkness. Low activity of linamarase, at the comparable level in light- and dark-grown calluses, was also observed, although dark-grown callus did not produce any cyanoglucosides. Shoots of 7-day-old seedlings, especially grown in light, showed about twice higher activity of linamarase than roots. However, in shoots regenerated, in vitro activity of linamarase was two times lower than that in roots regenerated under the same light conditions (Table 3).

Activity of HCN refixing β-CAS enzyme, much in the same way as linamarase, was about twice higher in light-grown shoots and roots than in dark-grown ones, both in seedling-derived and regenerated in vitro. On the other hand, there were no differences in β-CAS activity between dark- and light-grown callus tissues, as was observed for linamarase. Shoots isolated from 7-day-old seedlings in which the concentration of linamarin and lotaustralin was relatively high (in comparison to their concentration in roots) showed not only higher linamarase but also β-CAS activity. Inverse correlation was detected in organs of flax regenerated in vitro. Roots with lower than shoots concentration of cyanogens displayed higher activity of HCN refixing enzyme. It is worthy to note that in callus tissue with the low concentrations of linamarin and lotaustralin, and the low linamarase activity, β-CAS activity was 2–4 times higher (depending on organ) than in shoots and roots, both grown in dark and light conditions.

## Discussion

The presented data indicate that light positively affects organogenesis in hypocotyl-derived flax cultures. The stimulatory effect of light on direct in vitro organogenesis in oil-form flax was demonstrated before by Mundhara and Rashid (2002). Nevertheless, our results highlight dissimilar action of light and PGRs in regulation of these processes. Our data indicate that flax cultivar “Szafir” requires BA for induction of shoot organogenesis in darkness, and that light only enhances this process (Tables 1, 2). In contrast, Mundhara and Rashid (2002) demonstrated that regeneration of shoots in the presence of BA was strictly light dependent. These results suggest that depending on genotype, the media composition (mainly PGRs) for efficient organogenesis may vary. This phenomenon was also observed during indirect regeneration of transgenic flax (Caillot et al. 2009). Moreover, a stimulatory effect of light ( intensity of which was three times higher than that used in our study) on indirect organogenesis of flax was connected with its influence on the synthesis of photosynthetic pigments and the expression of genes encoding enzymes of the Calvin cycle (Caillot et al. 2009).

Our data also point to the beneficial effects of light on root organogenesis from hypocotyl explants (Table 2), phenomenon less known in flax in vitro. Its positive influence was rather described for shoots or seedlings rooting in some species, including flax (Bertazza et al.  1995; Tyburski and Tretyn 2004; Caillot et al. 2009).

Positive effect of light on organogenesis in flax in vitro, and in other species, e.g. *Lactuca sativa* L. (Kadkade and Seibert 1977), *Saccharum* *spp.* (Garcia et al. 2007), although commonly observed, seems not to be obligatory. In *Malus domestica* Borkh., for example, inhibition of both shoot and root organogenesis by light was detected (Liu et al. 1983). In contrast to our data (Table 1), formation of callus on some explants of *M. domestica* was even light-inhibited (Liu et al. 1983).

As indicated in our experiments, the rate of in vitro organogenesis in flax depended on light condition of donor plant, and was the most efficient on explants excised from light-growing seedlings, cultured under light conditions (Table 2). Some organogenetic processes, however, took place also in etiolated tissues, even in those from dark-grown explants. It indicates, that light perceived by the donor plant (flax seedling) during the growth phase preceding the in vitro culture, was not essential for a regenerative response of these explants. Light improved significantly tissue capability for organogenesis, similarly as it has been demonstrated for tomato (Bertram and Lercari 2000). In contrast, explants of *Oryza sativa* L. derived from dark-grown seedlings exhibited higher regeneration capacity as compared to light-grown ones (Pádua et al. 1998).

Shoots and roots of flax regenerated in vitro from hypocotyl explants and also light-grown callus accumulated cyanogenic glucosides linamarin and lotaustralin, which both are typical constituents of the intact plants (Niedźwiedź-Siegień 1998; Niedźwiedź-Siegień and Giersimiuk 2001) (Table 3). The production of cyanogenic compounds in callus is observed rather rarely e.g. was detected in *Schlechterina mitostemmatoides* (Passifloraceae), (Jäger et al. 1995), and in *Fagonia spp.* (Alam 2010). Osmotically stressed cell cultures of *Eschsoltzia californica* accumulated cyanogenic compounds, however, their composition were not identical to the one characteristic of the intact plants (Hösel et al. 1985). On the other hand, callus cultures of *Phaseolus lunatus* (Istock et al. 1990) or *Manihot esculenta* (Joseph et al. 1999) established from highly cyanogenic plant material were totally free of cyanogenic glycosides. Thus, the suggestion that biosynthetic pathway of cyanogenic glycosides may be absent or non-functional in dedifferentiated callus tissue is not valid for flax cultures, especially grown under light conditions. The apparent lack of linamarin and lotaustralin in dark-grown callus (Table 3) may be due to low content of these chemicals, below the determination threshold.

The concentration of cyanogenic glucosides in flax in vitro was the highest in shoots cultured under light conditions (Table 3). It may suggest their important role in plant stress physiology e.g. protection against herbivores (Zagrobelny et al. 2004). Positive effect of light on cyanoglucoside accumulation has been observed earlier in seedlings of flax (Niedźwiedź-Siegień and Giersimiuk 2001) and in other cyanogenic species (Nahrstedt et al. 1984; Burns et al. 2002; Vickery et al. 1987). It may result from regulatory effect of light on activities of enzymes involved in cyanoglucosides biosynthesis (Gaisser and Heide 1996). Light may also enhance the metabolic pools of valine and isoleucine, precursors of linamarin and lotaustralin, respectively.

The data presented in this article allow us to assume the attitude towards another hypothesis explaining the role of cyanogenic glycosides (or free HCN) in plants suggesting their involvement in some organogenetic processes (Selmar et al. 1988; Sanchez-Perez et al. 2009). HCN-p of the tissue depends on the concentration of cyanogenic glucosides, as well as activity of β-glycosidase (linamarase), which is the rate-limiting step in degradation of cyanogenic compounds (Kakes 1990; Vetter 2000). Both these parameters are elevated in light-regenerated organs, especially in shoots, in comparison with etiolated ones (Table 3). Consistently, differentiation processes completed by formation of exact organ are more efficient in light-grown cultures (Tables 1, 2). In contrast to Selmar et al. (1988) and Sanchez-Perez et al. (2009) postulating the nutritional function of cyanogenic glycosides in some organogenetic processes, we suggest that free HCN (but not cyanogens themselves), released from cyanogens may play a regulatory role. Therefore, low efficiency of regeneration in darkness may be correlated to weak HCN-p of these tissues (Tables 2, 3). On the other hand, higher activity of β-CAS in light-derived organs (Table 3) may indicate also that under these conditions the potential production of HCN may be higher than in darkness. Moreover, the light-HCN-p correlation observed in shoots of 7-day-old seedlings of flax (Table 3), suggests that some morphogenetic processes may be controlled in these organs by cyanide. In light-grown flax seedlings, the young leaves expanded earlier than in dark-grown ones (data not shown).

The signalling role of HCN in some processes has been described recently (Siegień and Bogatek 2006). Although the routes of HCN action are not well known, some data concerning its dormancy removal and stimulation of germination of some seeds indicate its interaction with other signalling molecules, e.g. ethylene, ROS and NO (Oracz et al. 2009; Gniazdowska et al. 2010). Moreover, it was detected that stress conditions which generate ROS and oxidative stress enhance differentiation of flax (Mundhara and Rashid 2001; Obert et al. 2005) and also other species (Szechyńska-Hebda et al. 2007).

Finally, in this study, the beneficial effect of light on regeneration of flax shoots and roots in vitro is underlined. Moreover, main data discussed above indicate that some organogenetic processes in flax in vitro may be under the control of HCN, production of which is also light-regulated in these tissues. However, additional experiments should be performed to clarify mode of cyanide action in plant organogenesis.

### **Author contribution**

The research topic and framework was defined, and the whole study supervised by I. Siegień. A. Adamczuk performed the tissue culture work and helped with interpretation of data. Determination of enzymes activities was carried out by K. Wróblewska. I. Siegień conducted the other experiments, wrote and revised the manuscript.

## Abbreviations

BA: 6-Benzyladenine
β-CAS: β-Cyanoalanine synthase
HCN: Hydrogen cyanide
HCN-p: Cyanogenic potential
KCN: Potassium cyanide
MS: Murashige and Skoog medium
NAA: α-Naphthalene acetic acid
NO: Nitric oxide
PGRs: Plant growth regulators
2,4-D: 2,4-Dichloro-phenoxyacetic acid
ROS: Reactive oxygen species
TLC: Thin layer chromatography
